# Mr. Fowler, on the Teeth

**Published:** 1800-01

**Authors:** William Fowler

**Affiliations:** Princes Street, Hanover Square


### *To the Editors of the Medical and Phyſical Journal.*

Gentlemen,

IF you think the two following caſes, objects worthy notice, I ſhall be gratified in ſeeing them placed in your very inſtructive Medical and Phyſical Journal.

I am, Gentlemen,
Your much obliged humble ſervant,
WILLIAM FOWLER.

*Princes Street, Hanover Square,*

*October* 12, 1799.

---

THE firſt of theſe caſes, is an inſtance of ſix teeth deſtroyed by the left canine tooth projecting acroſs the front of the palate, occupying the place of thoſe deſtroyed; and which long continued the undiſcovered cauſe of their deſtruction.

Case 2.—A gentleman whom I attended, was afflicted with the tooth-ach in the firſt dens molaris. Being much alarmed at the idea of extraction, he applied (unknown to me) to a Mrs. ———, who at that time was eſteemed famous for the cure of the tooth-ach without drawing. She had applied her noſtrum to the tooth, twice within the ſpace of three days; and on the fourth, he came to me, complaining of a ſore mouth, telling telling me, where he had been to get relief; and that the liquid which had been ufed was very cauftic. From the appearance of the violent inflammation, which had taken place from the difeafed tooth to the epiglottis, I advifed him to confult fome medical gentleman of eminence *immediately*; with which advice, I am forry to fay, he did not comply. Not hearing from him on the third day, I called (en paffant), but he was too ill to be feen; a derangement of intellects had taken place. I called again four days afterwards, and was informed, that he had died raving on the preceding day. I had every reafon to believe, that the fluid which had been inferted into the tooth, with a view of deftroying the nerve, had produced this tragical end.

---

